# Superior venous use of bridge balloon for percutaneous lead extraction in a patient with an interrupted inferior vena cava: a case report

**DOI:** 10.1093/ehjcr/ytag252

**Published:** 2026-04-15

**Authors:** Rea Ganatra, Curtis Page, Rebecca Preston, Christopher Aldo Rinaldi, Jonathan M Behar

**Affiliations:** Cardiovascular Department, St. Thomas’ Hospital, Guy’s and St. Thomas NHS Foundation Trust, Westminster Bridge Road, London SE1 7EH, UK; Cardiovascular Department, St. Thomas’ Hospital, Guy’s and St. Thomas NHS Foundation Trust, Westminster Bridge Road, London SE1 7EH, UK; Cardiovascular Department, St. Thomas’ Hospital, Guy’s and St. Thomas NHS Foundation Trust, Westminster Bridge Road, London SE1 7EH, UK; Cardiovascular Department, St. Thomas’ Hospital, Guy’s and St. Thomas NHS Foundation Trust, Westminster Bridge Road, London SE1 7EH, UK; Cardiovascular Department, St. Thomas’ Hospital, Guy’s and St. Thomas NHS Foundation Trust, Westminster Bridge Road, London SE1 7EH, UK

**Keywords:** Case report, Transvenous lead extraction, Bridge balloon, Adult congenital heart disease, Dextrocardia, Anomalous venous return, Interrupted inferior vena cava

## Abstract

**Background:**

Transvenous lead extraction (TLE) is the gold-standard treatment for cardiac implantable electronic device removal but carries a small risk of major complications, including superior vena cava (SVC) injury. The prompt use of endovascular occlusion devices can provide temporary haemostasis and haemodynamic stability in the rare event of SVC laceration. The presence of congenital heart disease and venous anomalies introduces additional technical complexity and necessitates careful procedural planning.

**Case summary:**

A 52-year-old gentleman with dextrocardia and situs inversus totalis was referred to our centre for transvenous lead extraction after having presented with pacemaker pocket erosion. A CT venogram confirmed a completely interrupted inferior vena cava (IVC) with azygos continuation. A Bridge Balloon was pre-emptively placed via a superior venous approach through the right internal jugular vein as the IVC interruption precluded conventional approach through the femoral vein. TLE was performed under general anaesthesia using laser and mechanical sheaths, with successful extraction of all three leads and no major complications. The patient underwent reimplantation of a dual-chamber ICD on the right side after completing antibiotic therapy and was discharged in good condition.

**Discussion:**

In IVC interruption, a superior approach for Bridge Balloon placement can provide effective protection against SVC injury. As survival of patients with congenital heart disease improves, awareness of anatomical variants and procedural adaptation is crucial for safe and successful lead extraction.

Learning points

- Venous anomalies are more common in patients with congenital heart disease, and pre-procedural imaging is essential in providing critical anatomical information when planning for transvenous lead extraction.
- A superior venous approach can enable safe deployment of an endovascular occlusion balloon in the absence of femoral access.

## Introduction

Transvenous lead extraction (TLE) is the gold standard approach for pacing or defibrillator lead removal when indicated. The rate of TLE has steadily increased in recent years in response to the dramatic rise in implantation of cardiac implantable electronic devices (CIED).^1^ Novel extraction techniques have emerged to improve the safety and efficacy of TLE, however there remains a 2%–3% risk of major complication.^2,3^ Injury to the superior vena cava (SVC), whilst uncommon, is a serious and potentially fatal complication of TLE. Prompt deployment and inflation of a percutaneously delivered Bridge Balloon (Phillips Inc.) at the site of SVC injury can achieve temporary haemostasis and haemodynamic stability, serving as a bridge to definitive open surgical repair. The bridging balloon is conventionally placed across the SVC, from an inferior approach via the femoral vein, most often with a stiff wire tip placed either in the internal jugular or subclavian vein.^4^

Infra-hepatic inferior vena cava (IVC) interruption with azygos continuation is a rare congenital anomaly often associated with heterotaxy syndrome or dextrocardia with or without situs inversus. IVC interruption has an estimated prevalence of up to 15% in patients with dextrocardia and poses an anatomical challenge for transcatheter based procedures.^5,6^ We report a successful high-risk TLE for a device erosion in a gentleman with dextrocardia, complete situs inversus and IVC interruption requiring superior venous access to pre-emptively site an endovascular occlusion balloon to protect the SVC. The procedure was undertaken at a high-volume tertiary referral centre performing approximately 700 device implantations and 75 extractions per year.

## Summary figure

**Figure:**
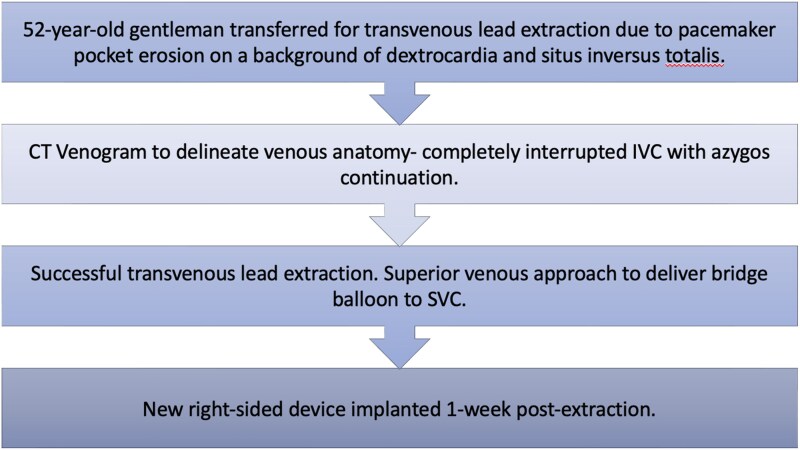


## Case presentation

A 52-year-old gentleman (height 167 cm, weight 61 kg, BMI 23 kg/m^2^) with a background of dextrocardia and situs inversus totalis was referred to our centre for transvenous lead extraction. His medical history included paroxysmal atrial fibrillation, ischaemic cardiomyopathy with prior myocardial infarction and severely impaired left ventricular function. He additionally had diagnoses of relapsing remitting multiple sclerosis and Crohn’s colitis.

He underwent dual chamber permanent pacemaker implantation for symptomatic sinus node disease in 2008 (Medtronic active fixation RA and RV leads). Due to increasing RV lead thresholds, he underwent a new RV lead implantation in 2015 (with the redundant RV lead capped and buried) alongside a generator replacement. A second generator replacement was undertaken in March 2025. Two months post generator replacement, there was evidence of redness and irritation overlying the device with subsequent device erosion. There were no features of systemic infection. Blood cultures obtained were negative. Wound swab cultures confirmed a light growth of Enterococcus Faecium which was thought to be a colonizing flora. Antimicrobial therapy was held in accordance with microbiology advice. A transthoracic echocardiogram confirmed normal left ventricular size with severely impaired systolic function (LVEF 30%–35%), consistent with his prior myocardial infarction. There was no significant valve disease and no obvious vegetation accepting the limitations of transthoracic imaging. A pacemaker check confirmed satisfactory lead parameters with an atrial pacing burden of 100% and 0.1% ventricular pacing.

**Figure 1:**
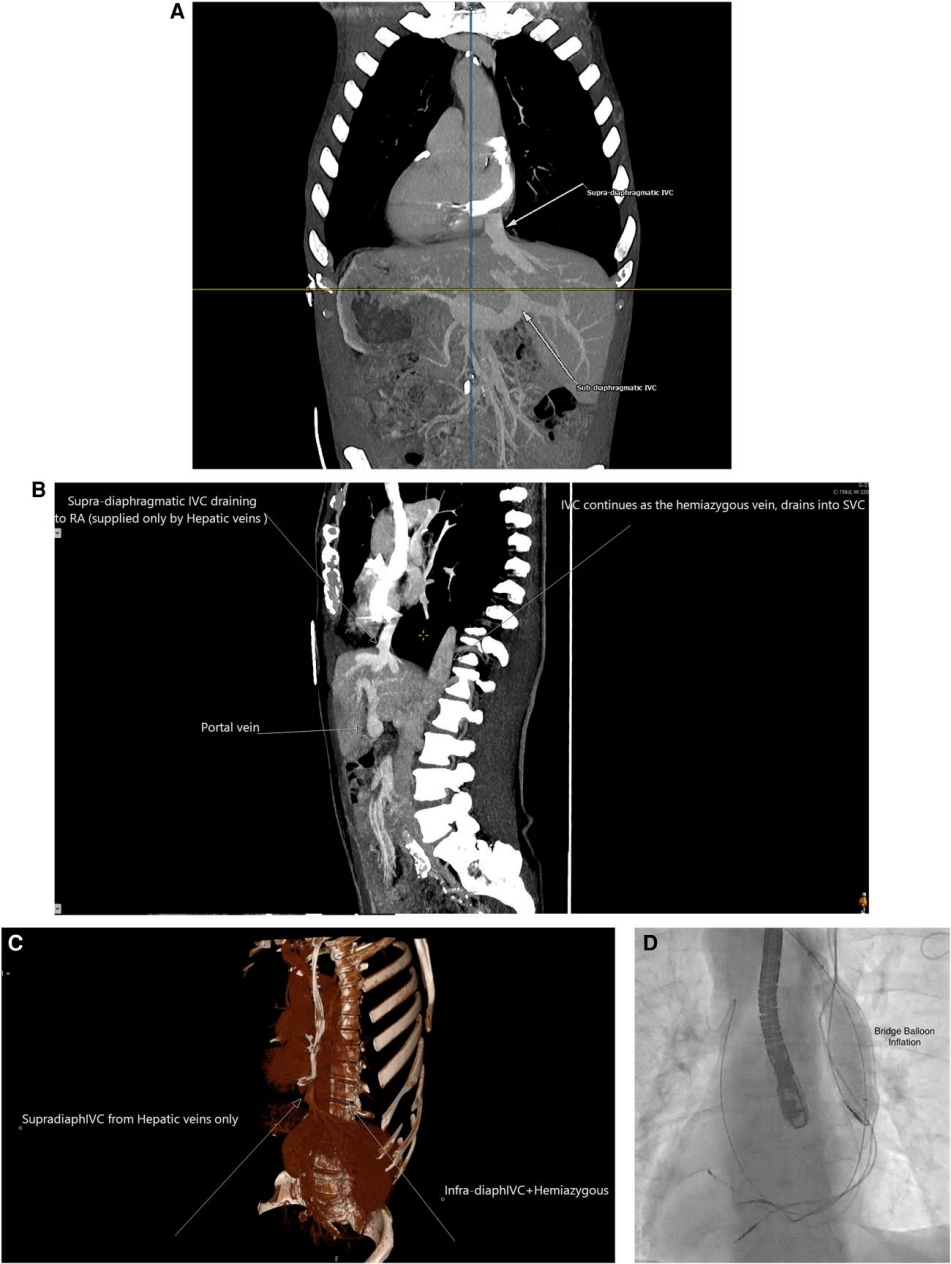
*(A-C)* Annotated computed tomography imaging demonstrating dextrocardia with interruption of the IVC. *(D)* Bridge Balloon delivered using superior venous approach.

Multi-disciplinary discussion confirmed a class I indication for percutaneous cardiac device system extraction. Given the association of congenital heart disease with vascular anomalies, a CT venogram was arranged to further delineate his venous anatomy. This confirmed a completely interrupted IVC which filled into a large, dilated azygous vein, draining superiorly into the innominate vein (*Figure 1*). The left internal jugular vein (IJV) was occluded and the right IJV appeared patent. The left SVC was severely stenosed distally at the point of entry of 3 leads into the right atrium.

Following a shared decision-making discussion, preparations were made for a lead extraction. The case was performed in a hybrid theatre under general anaesthesia using fluoroscopy and transoesophageal echocardiography (TOE). TOE provided continuous visualization for early intraoperative detection of pericardiac effusion in case of complication. Left and right sided venograms confirmed patent right central veins with occlusion of the left IJV as suggested on CT. RIJV access was obtained under ultrasound guidance following which two separate venous sheaths were placed (7Fr and 8Fr). A Terumo hydrophilic wire was advanced to the pulmonary artery and exchanged for a 6F Multipurpose angiographic catheter allowing passage of an Amplatz stiff guidewire and subsequent upgrade to a 12Fr sheath. A bridge balloon (Phillips) was placed into the SVC and inflated (*Figure 1*). During balloon inflation, a complete loss of systolic blood pressure was noted due to the interrupted IVC and the patient’s haemodynamic dependence on a superior venous return which was being temporary occluded. A temporary pacing wire was positioned in the right ventricle through the remaining RIJV sheath with good thresholds.

The pacemaker pocket was opened, and after disconnecting the generator, all three leads were mobilized up to the venous entry site. The active-fixation mechanisms were released from each lead, and LLD-EZY (Phillips) locking-stylets were deployed. Initial attempts to advance a 14Fr laser sheath over the newest RV lead were made however the acute angulation from the central veins into the SVC with calcified binding in the region between the SVC and RA precluded successful advancement. A 13Fr rotating mechanical sheath was subsequently deployed however due to extensive tightly bound length of calcification lead extraction remained challenging. Recurrent and sequential switching between laser and mechanical powered sheaths facilitated successful extraction of all three leads (*Figure 1*). TOE post-extraction confirmed no significant pericardial effusion. Venous access was maintained with a Terumo wire and long sheath and a temporary pacemaker implanted as he was pacing dependent. Total procedural time was 295 min.

A 7-day course of Flucloxacillin 500 mg four times a day was completed, and he was reimplanted with a new device from the right side. Given the background of severe ischaemic cardiomyopathy in addition to requirement for predominantly atrial pacing, a dual chamber transvenous ICD was implanted and he was subsequently discharged home. Clinical follow up at the 6-week check showed satisfactory lead measurements with no events seen on interrogation.

## Discussion

Guideline and expert consensus statements from the Heart Rhythm Society, American Heart Association/American College of Cardiology, and the European Heart Rhythm Association provide a Class 1 recommendation for total system extraction for the management of pocket infection (with or without erosion), CIED-related endocarditis and occult bacteraemia.^7,8^ When indicated, percutaneous transvenous lead extraction is considered the preferred strategy over a surgical approach. Whilst percutaneous advancements have improved the safety profile of TLE, major complications predominantly involving the venous vasculature or myocardium may still arise.

Anomalies of systemic venous return represent a heterogenous group of malformations and may occur in isolation or in the presence of other congenital heart disease. Interruption of the IVC is a particularly rare congenital anomaly with an incidence of 0.3% in the general population which increases up to 2% in those with other congenital heart defects. Their presence may make both implantation and extraction procedures more challenging. Procedural planning and CT are critical in providing additive anatomical information in this setting. To the best of our knowledge, this is the first description of placement of a bridge balloon via a superior venous approach to protect the SVC. In the setting of IVC interruption, delivering the Amplatz guidewire to the main pulmonary artery is a suitable and stable landing zone for the J tip of the wire.

Here, dextrocardia with situs inversus totalis provided an additional anatomical challenge. Whilst not specifically used in this case, left-right inversion of fluoroscopic imaging intra-procedurally can be useful to simulate levocardia and facilitate the operator’s anatomic orientation. With approximately 90% of patients with congenital heart disease now surviving into adulthood, the likelihood of encountering individuals with ACHD is greater and cardiologists must be equipped to manage these cases.

## Lead author biography


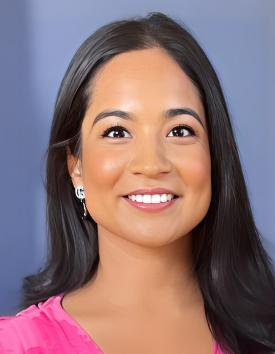


Dr Rea Ganatra obtained her medical degree in 2016 from Barts and the London School of Medicine and Dentistry. She is a cardiology registrar in the South London deanery, and is currently out-of-programme completing a clinical and research fellowship in structural cardiology at Guy's and St. Thomas’ NHS Foundation Trust.

## Supplementary Material

ytag252_Supplementary_Data

## Data Availability

The data underlying this article are available in the article and in its online Supplementary material.

## Supplementary material

**Consent:** Patient’s consent was obtained in compliance with the COPE guidelines.
