# Supplementary material for: Superior venous use of bridge balloon for percutaneous lead extraction in a patient with an interrupted inferior vena cava: a case report
Source: Eur Heart J Case Rep. 2026 Apr 15;10(4):ytag252. doi: 10.1093/ehjcr/ytag252 (PMC13128198; doi:10.1093/ehjcr/ytag252)
Supplement: ytag252_Supplementary_Data [file ytag252_supplementary_data.zip › EHJ video clips.pptx]

## Slide 1
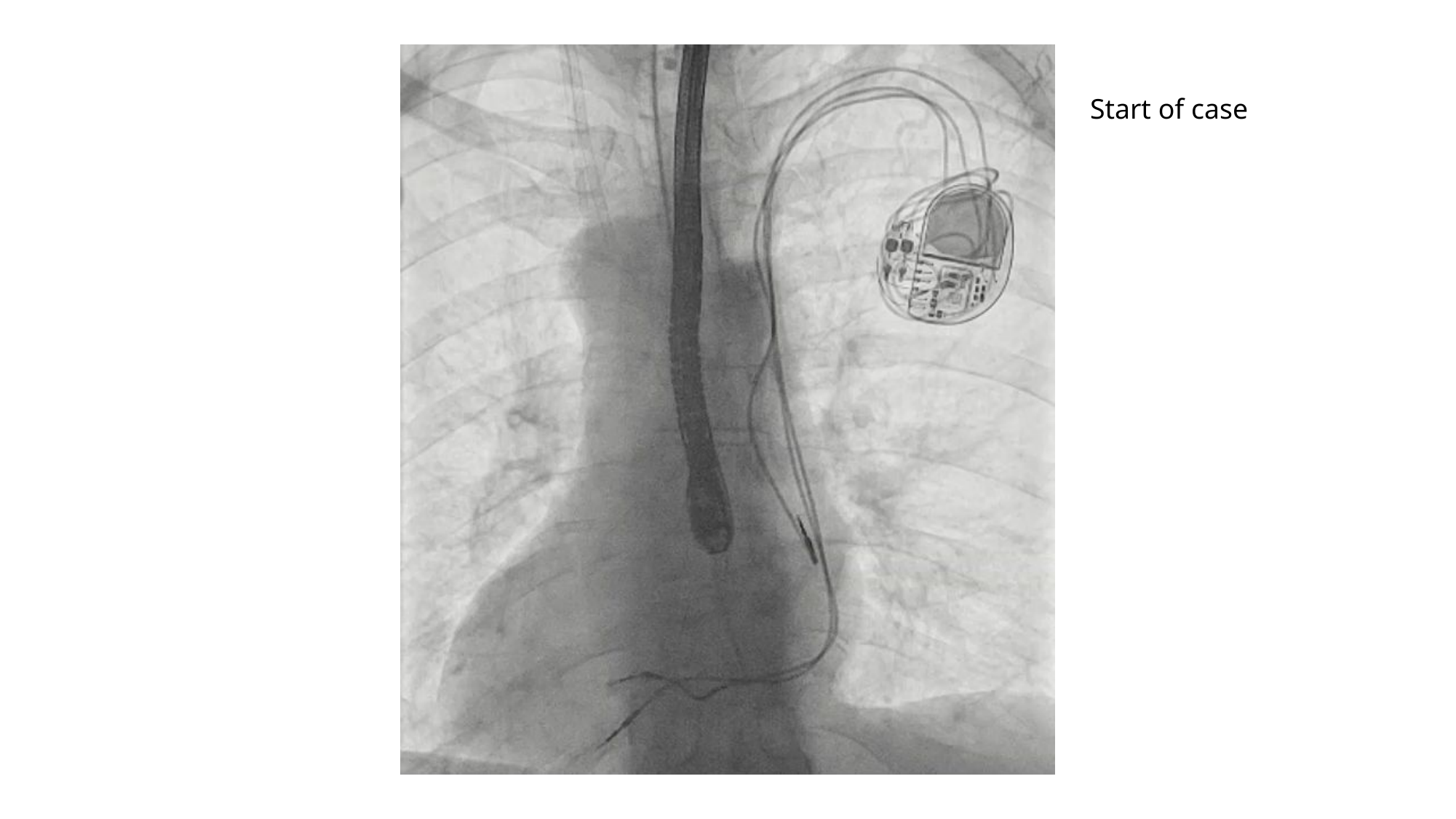

Start of case

## Slide 2
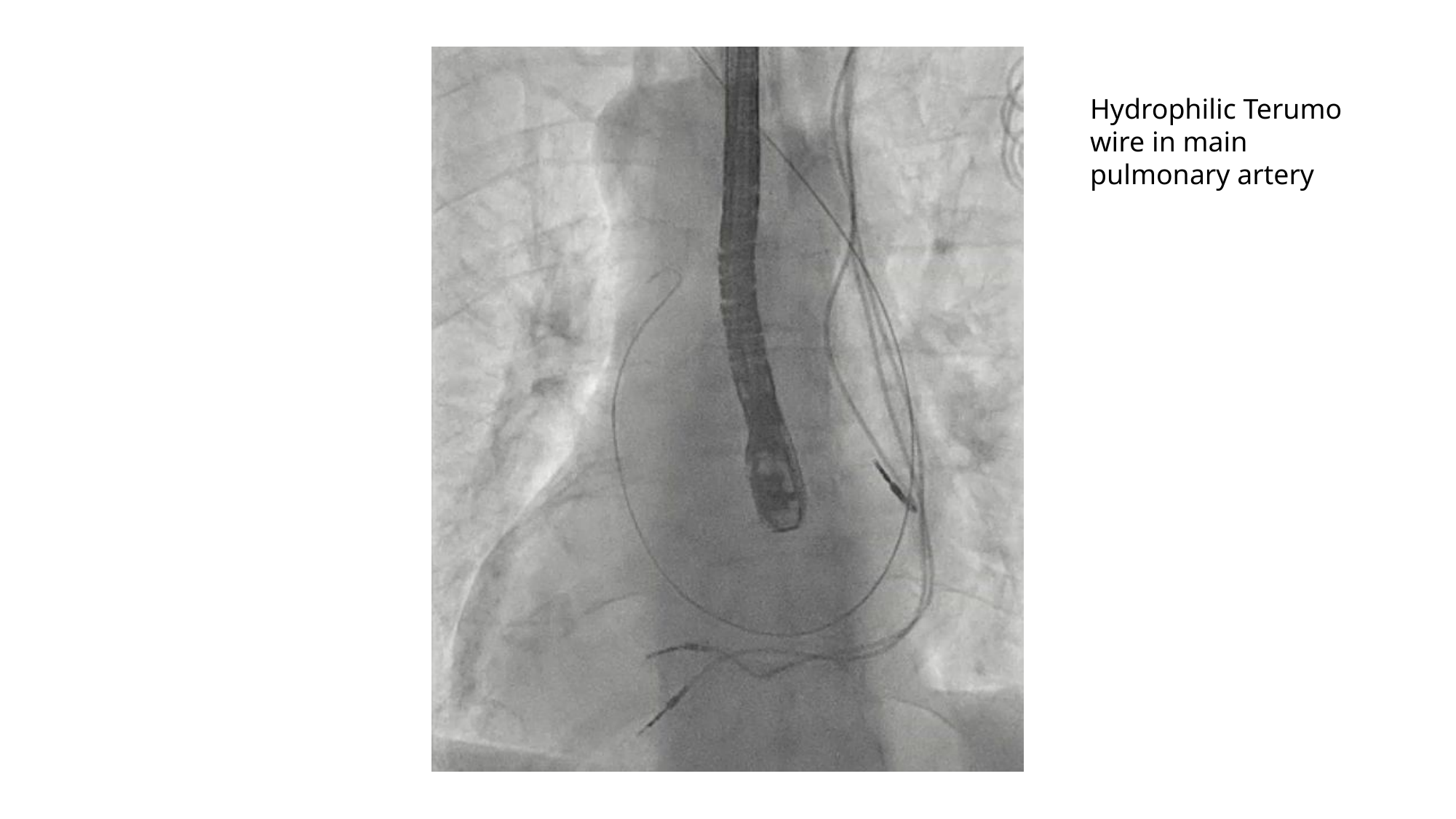

Hydrophilic Terumo wire in main pulmonary artery

## Slide 3
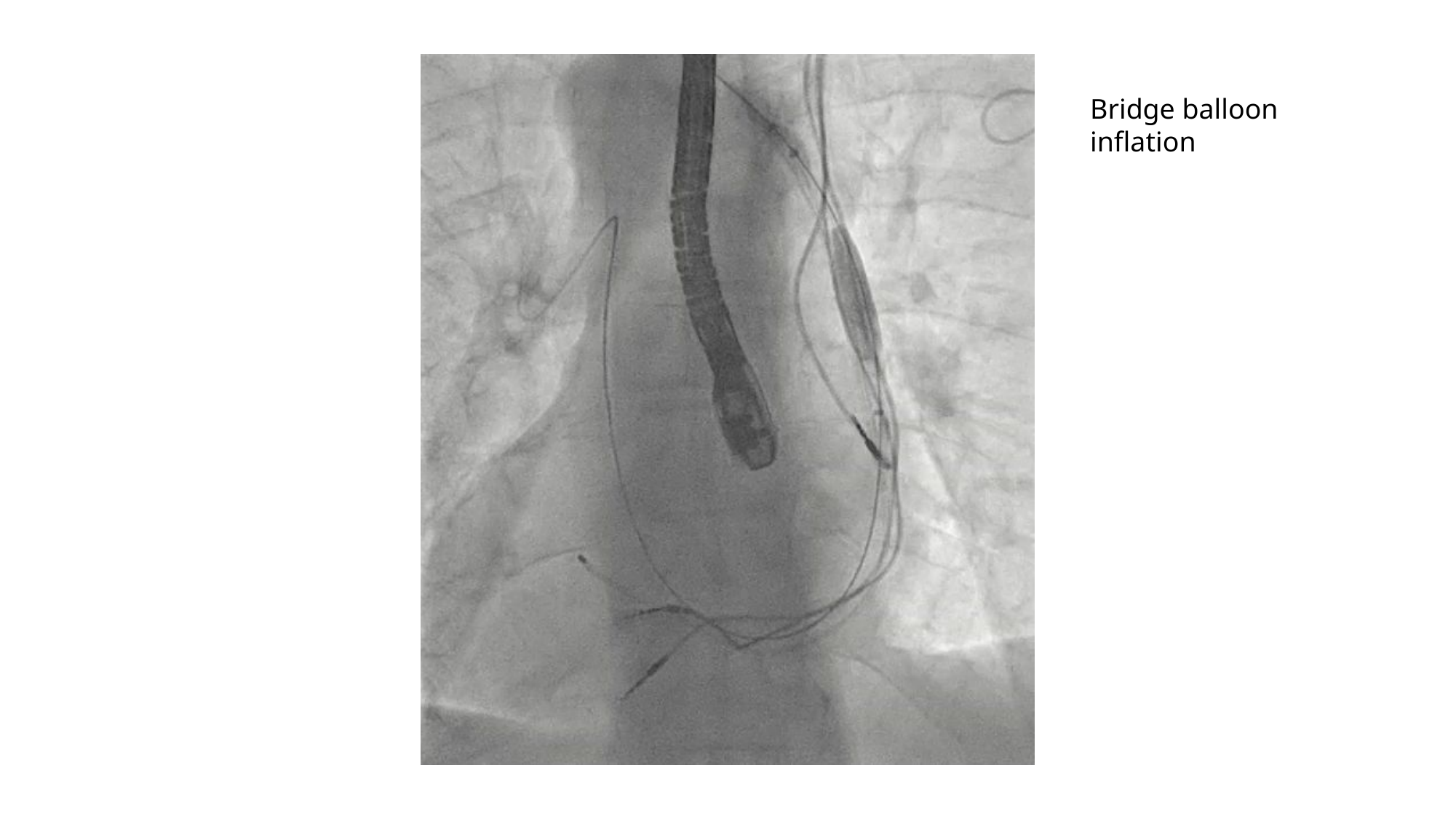

Bridge balloon inflation

## Slide 4
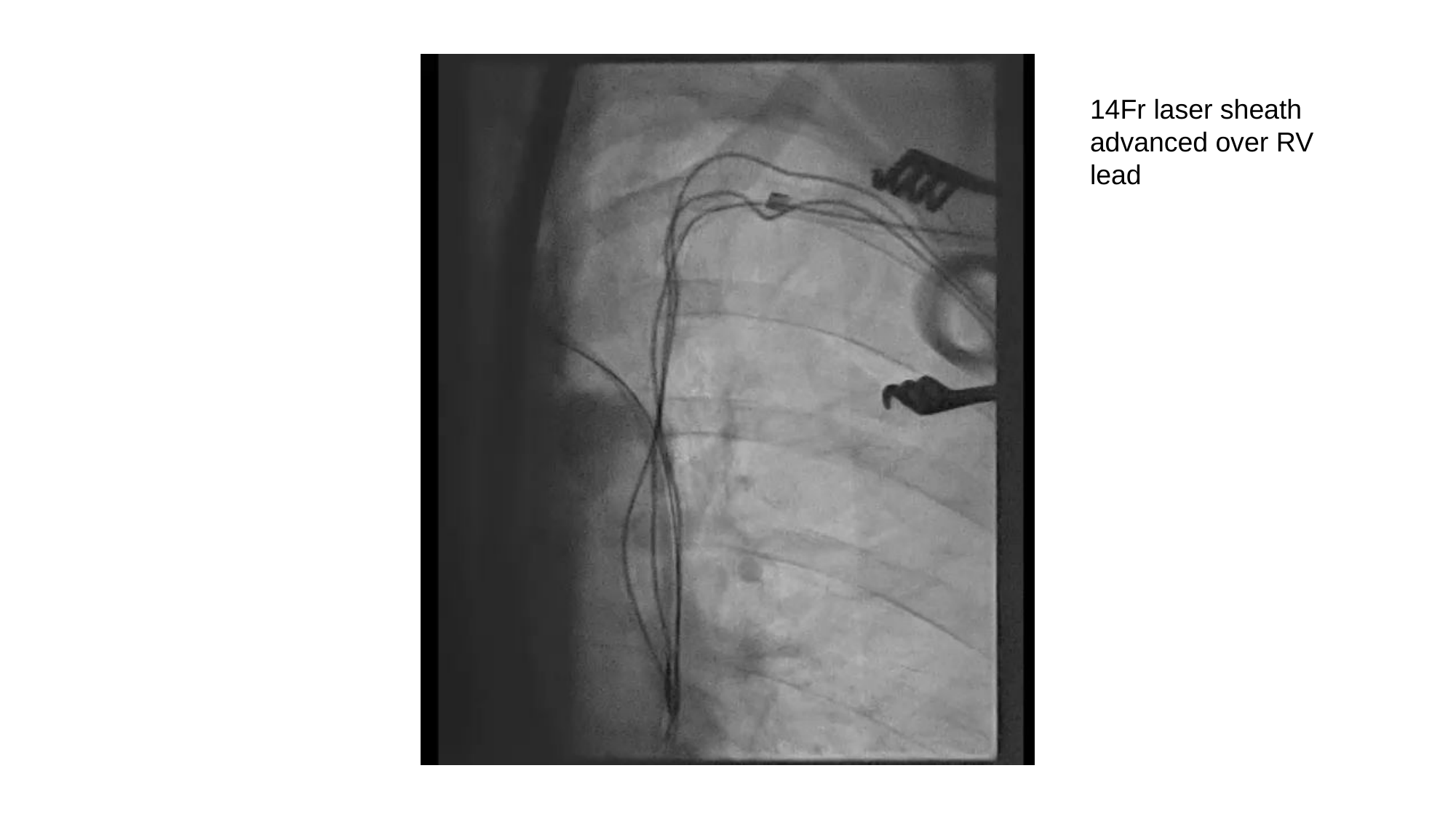

14Fr laser sheath advanced over RV lead

## Slide 5
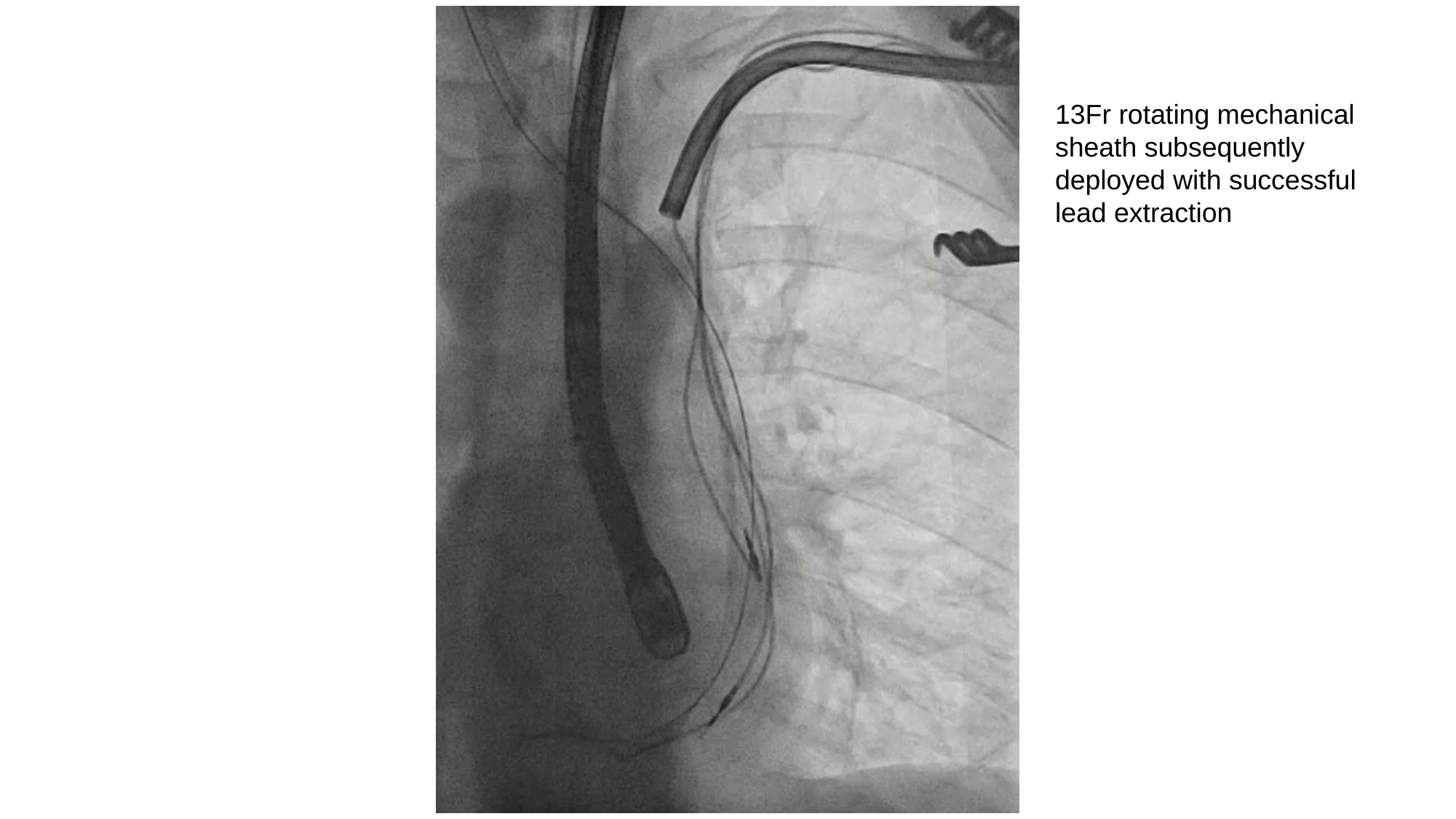

13Fr rotating mechanical sheath subsequently deployed with successful lead extraction
